# Patient-ventilator asynchrony during conventional or automated pressure support ventilation in difficult-to-wean patients

**DOI:** 10.1186/cc10733

**Published:** 2012-03-20

**Authors:** MM Bitondo, HM Aguirre-Bermeo, A Moccaldo, P De Santis, V Bernini, A Tersali, S Italiano, DL Grieco, FA Idone, J Grandjean, F Roche-Campo, M Antonelli, J Mancebo Cortes, SM Maggiore

**Affiliations:** 1 Catholic University of the Sacred Heart, Roma, Italy; 2 San Pau University Hospital, Barcelona, Spain

## Introduction

Patient-ventilator asynchrony, defined as a mismatch between patient's inspiratory time and the ventilator insufflation time, occurs in nearly 25% of intubated patients. High asynchrony rates are associated with higher incidence of weaning failure and tracheostomy, and prolonged mechanical ventilation. The aim of this study was to compare the asynchrony rate during conventional pressure support ventilation (PSV) and automated PSV (SmartCare; Draeger) in difficult-to-wean patients.

## Methods

A prospective, crossover study in difficult-to-wean patients (patients who required up to three spontaneous breathing trials (SBTs) or as long as 7 days to achieve successful weaning). Patients were ventilated with an Evita XL ventilator for two consecutive 3-hour periods applied in random order: with conventional PSV managed by the attending physicians; and with PSV managed by SmartCare. The periods were administered in the afternoon (3:00 to 9:00 pm) and in the night (12:00 pm to 6:00 am). In both periods, the starting PS level with either conventional or automated PSV was the basal level before enrolment. During every period, airway pressure, flow and volume signals were continuously recorded on a PC connected to t he ventilator using dedicated software (VentView). These signals were analyzed offline by two clinicians. The asynchrony index was defined as the number of asynchronies (wasted efforts, double cycles, premature cycling off ) divided by the total respiratory rate (ventilator cycles + asynchrony events), multiplied by 100.

## Results

Sixteen patients were enrolled (age 64 ± 11 years; SAPS II 66 ± 14; COPD 25%; days of mechanical ventilation before enrollment 9 ± 4, number of SBTs 3 ± 1). The asynchrony index was lower with Smartcare (10% vs. 14%, *P *= 0.01), but not different between afternoon and night. Mean PS level (11 vs. 12 cmH_2_O) was not different between conventional and automated PSV, although the coefficient of variability of PS level was greater with Smartcare (20% vs. 0%, *P *< 0.01). No differences were observed in PaCO_2 _(36 vs. 36 mmHg), PaO_2 _(106 vs. 102 mmHg), total respiratory rate (22 vs. 23), and P0.1 (1.4 vs. 1.6 cmH_2_O) between conventional PSV and Smartcare.

## Conclusion

As compared with conventional PSV, Smartcare may reduce asynchronies in difficult-to-wean patients, possibly because of greater variability of the PS level. This needs to be further confirmed.

